# Patient–clinician communication research for 21st century health care

**DOI:** 10.3399/bjgp22X718277

**Published:** 2022-01-28

**Authors:** Jackie van Dael, Alex Gillespie, Ana Luísa Neves, Ara Darzi

**Affiliations:** National Institute for Health Research (NIHR) Imperial Patient Safety Translational Research Centre, Institute of Global Health Innovation, Imperial College London, London, UK.; Department of Psychological and Behavioural Science, London School of Economics, London, UK; Bjorknes University, Oslo, Norway.; NIHR Imperial Patient Safety Translational Research Centre, Institute of Global Health Innovation, Imperial College London, London, UK.; NIHR Imperial Patient Safety Translational Research Centre, Institute of Global Health Innovation, Imperial College London, London, UK.

## INTRODUCTION

The pandemic has accelerated the shift to the unchartered waters of virtual healthcare delivery. Unprecedented numbers of consultations are now held remotely, introducing a range of novel challenges and uncertainties. One major area of concern relates to potential impacts on patient–clinician communication. Research on this has been limited so far, but early studies have indicated that there are critical differences between how patients and clinicians interact over telephone or video compared to face to face. These include patients raising fewer issues, clinicians seeking less information, and less psycho–social talk.^1^

The rapid development of a strong evidence base is urgently needed to understand if, when, and how communication is affected. With this objective in mind, we discuss major lines of inquiry in patient–clinician communication research, and argue why theoretical and methodological approaches from social sciences and linguistics will be particularly important in supporting future endeavours to identify and mitigate risks associated with virtual consultations.

## DIFFERENT PARADIGMS IN PATIENT–CLINICIAN COMMUNICATION RESEARCH

Despite decades of research, patient–clinician communication remains imprecisely defined and operationalised in health research.^2^ A large area of work has focused on clinician (and patient) skills and behaviours. Adopting umbrella constructs such as ‘patient-centred communication’ and ‘shared decision making’, these studies measure certain observable communication behaviours (such as information giving and socio-emotional talk) using observational coding and rating scales. Through such quantification, this strand of research has enabled the identification of associations between patterns of communication and critical outcomes (for example, satisfaction, malpractice claims, and adherence).^3^^,^^4^

Yet, while such research will continue to be important for understanding systematic differences in content and outcomes between consultation modes, they must be accompanied by a focus on the interactional, situated, and process-like functioning of communication.

Deviating from popular methods in mainstream health research, which tend to be informed by a positivist epistemology, communication research in dominant paradigms of (socio)linguistics and social sciences examine communication as a dynamic, interactional process that is simultaneously shaped by both interactants, the context in which it takes place, and wider sociocultural norms and rules.^5^ As these fields recognise that people rely on context to interpret the meaning of talk, and can have very different interpretations of the same words, methods tend to focus on the processes through which patients and clinicians create and maintain shared understanding through multi-turn communication: using the interaction, rather than the individual, as the unit of analysis.

As we argue below, the operationalisation of communication as a situated, social process will be essential to identify and mitigate potential sources of miscommunication associated with telephone or video consults, and to understand how talk functions in virtual context.

## RESEARCH PRIORITIES FOR PATIENT–CLINICIAN COMMUNICATION IN VIRTUAL CONSULTATIONS

### From isolated behaviours to situated interactions

Behavioural approaches to communication do not give insight into problems at the interpersonal level (such as misunderstandings and missed information), which remain a problem in traditional care, and are expected to occur at an even greater rate in virtual consultation modes. Despite considerable investment in clinician competency and skills, studies consistently show that clinicians frequently overestimate the patient’s understanding of their advice and often do not suspect miscommunication has occurred, resulting in missed opportunities to mitigate their communication accordingly.^6^^,^^7^ These problems are likely greater in video or telephone consultations where the context is less shared (for example, the clinician and patient are in a different spatial location), the channels for communicating are reduced (for example, missing non-verbal cues), and signals of communication problems (such as hesitations, vagueness, agitation, and silence) may be harder to detect and interpret (for example, due to transmission delays and absence of non-verbal cues). Indeed, a recent study found that latency in video consultations can result in misinterpretations of silences and gaze behaviour, resulting in misplaced practices for resolving conversational issues.^8^

While popular coding methods, such as the Roter interaction analysis system,^9^ are critical for understanding systematic differences in observable behaviour between consultation modes, they give little indication of the success of those behaviours in terms of whether a shared understanding was achieved. This is significant as the quality of mutual comprehension will in many cases be a mediator between communication patterns and distal outcomes such as treatment adherence or disease self-management.^3^ Similarly, rating scales can help judge clinician behaviours against a predefined standard but do not provide insight into whether these behaviours were adaptive in the consultation’s specific context, or how it was interpreted by the patient. While patients and clinicians agree on the components that comprise ‘good communication’ (such as information seeking and socio-emotional communication), they often disagree on whether these skills are present in a consultation.^10^

To capture relational discrepancies between patients and clinicians, we require methods that focus on the interactive and procedural nature of communication: how patients and clinicians simultaneously contribute to shaping interaction sequences (for example, through responding and turn-taking), how they negotiate their potentially differing individual (or cultural) interpretations of what is said, and how this is interdependent with the conversation’s (in)direct context. By adopting the interaction (rather than behaviour) as the unit of analysis, dialogical methods (such as interpersonal perception methods and conversation analysis) can offer insight into how misunderstandings emerge and can be resolved, bringing into focus reciprocal behaviours, such as mutual checking, verifying, and repairing, to secure and maintain a mutual understanding of issues discussed. If misunderstandings are indeed more likely to occur in video or telephone consultations, then discovering and repairing misunderstandings through dynamic and interactive approaches becomes all the more important.

The increased adoption of virtual care also represents a major opportunity for advancing this field, which is distinctive in requiring naturally occurring data, with minimal interference from researchers. The operational challenge of obtaining such data has historically limited progress, but with unprecedented rates of consultations now held virtually there is an imminent opportunity to advance this body of work.

### Towards contextual and multilevel approaches

A further consideration, especially critical for virtual healthcare delivery, is the need to adopt methods that allow for a broader understanding of how communication interrelates with context at multiple levels. This includes direct factors, such as patient and clinician characteristics (for example, language proficiency and clinician expertise), their interaction (for example, race/gender concordance and quality of their relationship), and their surrounding environment (such as characteristics of the consultation mode). It is evident that technical factors in virtual consultations, such as latency, sound or camera quality, lower consultation length, or transmission delays are fundamental. Other non-technical factors, such as language proficiency, degree of privacy, extent of shared background information, or the presence of health advocates (such as relatives and caregivers), may also play a greater or different role in online platforms compared to face-to-face consultations, and can introduce new challenges to equity in the quality of care. The sudden widespread adoption of virtual care also represents a major shift in indirect macro-level structures (for example, organisation of health delivery, media, and power relations), which have remained somewhat understudied in communication research.^11^ Neglecting these contextual dimensions, or treating them in isolation, risks leaving novel hazards associated with remote care undetected.

Studies that combine ethnography and analysis of talk to gain a contextual understanding of patient–clinician interactions remain relatively underrepresented, but are growing in number, and are a promising development towards detecting, preventing, and resolving conversational problems associated with virtual modes. For example, a recent study identified a number of conversational strategies to address interactional challenges that occur in video consultations, such as acts of repeating and paraphrasing to prevent potential loss of information due to audio or video glitches.^12^ Similarly, an ethnomethodological study on video consultations by Pappas and colleagues highlighted the centrality of the opening phase in video consultations as a moment of negotiating roles and structuring talk.^13^ Future work requires wider investment in such interactional and context-sensitive approaches to communication across a range of circumstances to ensure safe and high-quality virtual care.

## CONCLUSION

Meeting the challenges posed by healthcare’s digital transformation demands rapid investment in multidisciplinary research to ensure novel risks are identified, prevented, and mitigated. Studies of patient–clinician communication that adopt an interactional and multifaceted approach will have ever greater importance. The shift to virtual consultations also represents a huge opportunity for advancing the field of patient–clinician communication. Never before have consultations via audio and video platforms been implemented at such a large scale, offering a low-cost and feasible means to capturing and analysing naturally-occurring patient–clinician interactions across different contexts, settings, and groups. If studied appropriately, this can fundamentally recalibrate and enrich our understanding of patient–clinician communication, its functioning across different contexts, and how it can be improved.
